# The bacterial community of the lone star tick (*Amblyomma americanum*)

**DOI:** 10.1186/s13071-020-04550-z

**Published:** 2021-01-14

**Authors:** L. Paulina Maldonado-Ruiz, Saraswoti Neupane, Yoonseong Park, Ludek Zurek

**Affiliations:** 1 Department of Entomology, Kansas State University, Manhattan, KS USA; 2 Central European Institute of Technology, Center for Zoonoses, University of Veterinary and Pharmaceutical Sciences, Brno, Czech Republic; 3 Department of Chemistry and Biochemistry, Mendel University, Brno, Czech Republic

**Keywords:** Microbiome, Bacterial diversity, Midgut, Culturing, High throughput sequencing

## Abstract

**Background:**

The lone star tick (*Amblyomma americanum*), an important vector of a wide range of human and animal pathogens, is very common throughout the East and Midwest of the USA. Ticks are known to carry non-pathogenic bacteria that may play a role in their vector competence for pathogens. Several previous studies using the high throughput sequencing (HTS) technologies reported the commensal bacteria in a tick midgut as abundant and diverse. In contrast, in our preliminary survey of the field collected adult lone star ticks, we found the number of culturable/viable bacteria very low.

**Methods:**

We aimed to analyze the bacterial community of *A. americanum* by a parallel culture-dependent and a culture-independent approach applied to individual ticks.

**Results:**

We analyzed 94 adult females collected in eastern Kansas and found that 60.8% of ticks had no culturable bacteria and the remaining ticks carried only 67.7 ± 42.8 colony-forming units (CFUs)/tick representing 26 genera. HTS of the 16S rRNA gene resulted in a total of 32 operational taxonomic units (OTUs) with the dominant endosymbiotic genera *Coxiella* and *Rickettsia* (> 95%). Remaining OTUs with very low abundance were typical soil bacterial taxa indicating their environmental origin.

**Conclusions:**

No correlation was found between the CFU abundance and the relative abundance from the culture-independent approach. This suggests that many culturable taxa detected by HTS but not by culture-dependent method were not viable or were not in their culturable state. Overall, our HTS results show that the midgut bacterial community of *A. americanum* is very poor without a core microbiome and the majority of bacteria are endosymbiotic.
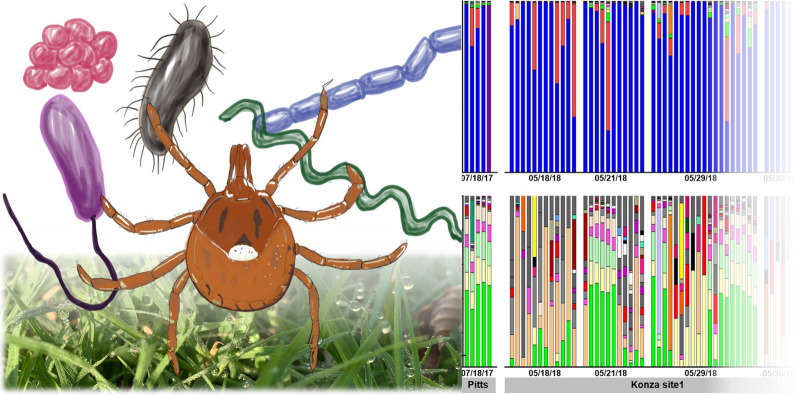

## Background

Hard ticks (Ixodidae) are among the most important arthropod vectors of human and animal pathogens in the US and worldwide [1–3]. The lone star tick (*Amblyomma americanum*) is common in the Midwest and Eastern USA [4] and an important vector of *Fransicella tularensis*, *Ehrlichia chaffeensis*, *E. ewingii*, and heartland virus [5–7]. This tick is also known to cause red meat allergy because of alpha-gal in its salivary glands [8, 9]. In addition to pathogens, lone star ticks carry a commensal and symbiotic bacterial community [10], which may play a role in the vector competence for pathogens [11, 12] although this role remains to be elucidated [12]. Most studies on the microbiome of *A. americanum* focused on intracellular endosymbionts [13–16] although extracellular bacteria in the gut lumen may influence the colonization of pathogens [10, 12, 17] and overall vector competence of ticks [10, 11]. Using culture-independent approaches, earlier studies have shown great microbial diversity in the lone star tick, reporting several hundreds of operational taxonomic units (OTUs) with high alpha diversity and at least 99 bacterial families and over 100 genera [18–20].

Interestingly, most culture-independent microbiome studies on the tick gut report bacterial communities that should be easily culturable, such as *Bacillus*, *Pseudomonas*, *Escherichia*, *Shigella*, and *Proteus* [10, 21, 22], and therefore should be coupled with a culturing approach to determine the abundance of viable bacterial taxa. These culturable bacterial isolates might then become available for future studies on manipulation of the gut bacterial community and its effect on the tick vector competence. In this study, we aimed to survey the microbiome of *A. americanum* using the parallel culture-dependent and culture-independent approaches.

## Methods

### Field sites, tick collection, and sample preparation

Adult females of *A. americanum* (*n* = 120) were collected from northeastern Kansas (Konza Prairie Biological Research Station: 39°06′23.4″ N, 96°36′11.4″ W and 39°06′16.6″ N, 96°35′43.7″ W) and southeastern Kansas (Pittsburg Wilderness Park 37°27′09.8″ N, 94°42′41.0″ W) by flagging. Ticks were placed in a cooler with high humidity (> 90% RH) and transported to the laboratory. Ticks were surface sterilized upon arrival using 0.5% sodium hypochlorite (5 min) and 70% ethanol (3 min) and washed three times with sterile water. The mouthparts and anus of ticks were sealed with a glue (SuperGlue, Pacer Technology, Inc., CA, USA) to prevent access of chemicals to the gut lumen during sterilization. Then, individual ticks were immobilized on a sterile wax surface and aseptically dissected in phosphate-buffered saline (PBS; MP Biomedicals, LLC, CA, USA) to remove soft tissues (midgut, salivary glands, and ovaries). Tissues from each individual tick were homogenized in PBS at a total volume of 200 µl and divided into two equal parts. One half (100 µl) was immediately used for culturing, and the other half was stored at − 80 °C for DNA extraction and culture-independent analysis. These homogenates were analyzed individually and were recorded for each individual tick.

### Culture-dependent method

**Fig. 1:**
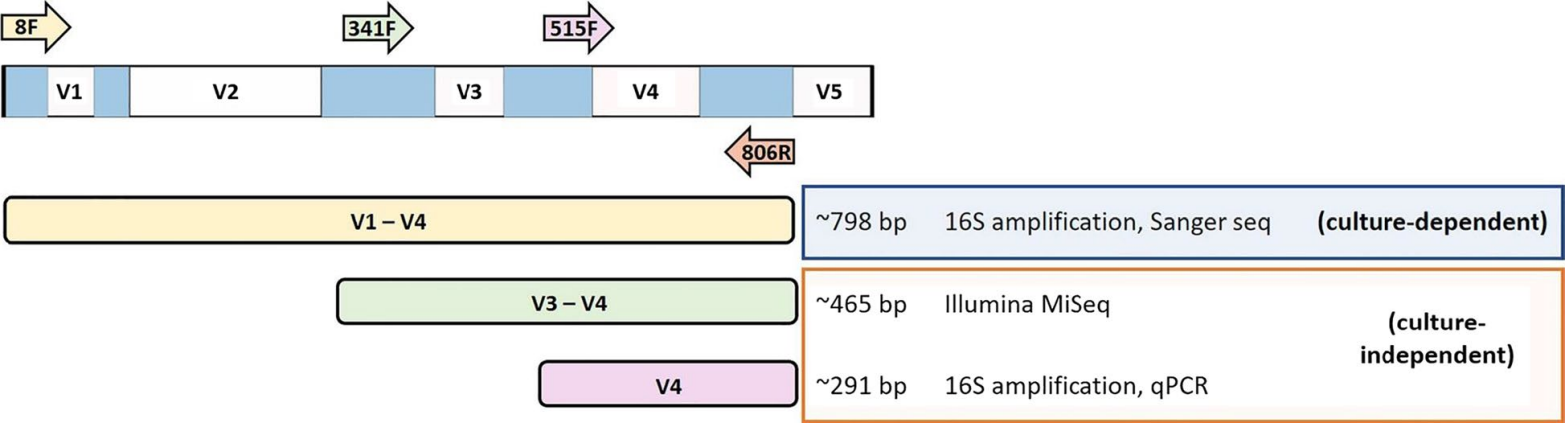
Schematic representation of primers in the hypervariable regions (V1–V5) of the 16S rRNA gene for Sanger sequencing, high throughput sequencing, and quantitative PCR

Tissue homogenates (100 µl) were serially diluted in PBS spread plated on trypticase soy agar (TSA) (BD, Sparks, MD, USA) and incubated at 25 and 37 °C for 72 h in aerobic and microaerophilic (CampyPakPlus^TM^ GasPak^TM^ system jars, BD, BBL^TM^ Franklin Lakes, NJ, USA) conditions. Colony-forming units (CFU) per tick of each distinct colony morphology were counted and calculated in CFU per tick. Morphologically distinct colonies were sub-cultured on TSA for characterization and identification. Rapid tests for catalase activity using hydrogen peroxide and gram test using potassium hydroxide were conducted. DNA extraction was performed with the ZymoBiomics DNA Miniprep kit (Zymo Research, CA, USA). The 16S rRNA gene was amplified using universal bacterial primers for the V1–V4 regions (8F and 806R) [23] (Fig. 1) and sequenced by the Sanger method. Sequences were edited, aligned, and phylogenetically analyzed in MEGA-X (Molecular Evolutionary Genetics Analysis version 10.1.6). Alignment was generated using the multiple sequence comparison by log-expectation (MUSCLE) and the UPGMA clustering methods. The tree was generated by the maximum likelihood (ML) method with 500 bootstrapping. Taxonomic affiliation of each sequence was determined using the Basic Local Alignment Search Tool (BLASTn) at the NCBI GenBank database [24] and verified through the Ribosomal Database Project (RDP) classifier, using the sequence matching tool [25, 26].

### Culture-independent approach

A total 94 out of 120 ticks were randomly selected for sequencing. The total DNA from individual homogenates was extracted with the ZymoBiomics® DNA Miniprep kit (Zymo Research, CA, USA) following the manufacturer's protocol. As control we used sterile deionized water for sample preparation containing DNA from *E. faecalis* V583 extracted using the same DNA extraction protocol as that for ticks. The DNA was quantified using a spectrophotometer (NanoDrop 2000, Thermo Scientific, USA) and a fluorometer (PicoGreen, Invitrogen, MA, USA). To assess the quantity of 16S rDNA, serial dilutions of a known amount of tick DNA were used for quantitative PCR (qPCR) standardization curves using the single copy gene of the tick *V-ATPase subunit C*. We used similar DNA template amounts (between 5 and 20 ng across samples) for both genes in qPCR. The qPCR reaction was prepared using the 2× Luna® Universal qPCR Master mix following the manufacturer's instructions. A single copy gene from *A. americanum* (*V-ATPase* subunit *C*) representing the quantity of tick chromosome, was used to normalize the bacterial 16S copy number. (primers: 894F: 5′-CCC TGA GGC TTT TTG TTG AG-3′ and 1043R: 5′ CCT GGG CAA TGC TTG TGT-3′). For quantification of the 16S rRNA gene, the V4 region amplification with universal eubacterial primers 515F: 5′-GTG YCA GCM GCC GCG GTA A-3′ and 806R: 5′-GGA CTA CNV GGG TWT CTA AT-3′ (modified from Caporaso et al. [27]) (Fig. 1) was used. Delta Ct values were calculated by the difference in qPCR Ct values of the 16S rDNA and the tick V-ATPase subunit C.

Library preparation and sequencing of the V3 and V4 region of the 16S rRNA gene (341F and 806R) (Fig. 1) were performed at the Genome Sequencing Core of the University of Kansas. Libraries were generated using unique dual indexing (UDI) and prepared using the Nextera XT index kit. Sequencing was conducted using the MiSeq Next Generation Sequencer. Raw sequence reads were analyzed using the Mothur software package (version 1.39.5, [28]). Paired-end sequences for 300 nt were joined, and sequence reads with low quality (*q* < 25), ambiguous base, and ambiguous length (< 100 and > 450 bp) were removed. All sequences other than that of *E. faecalis* from the positive control sample were also filtered out. High-quality sequences were aligned with SSU rRNA SILVA reference alignment [29] using the Needleman-Wunsch global alignment algorithm [30]. Chimeric sequences were checked using UCHIME [31] and removed. Non-*E. faecalis* sequences from the positive control sample were also removed. Sequence reads were then clustered into OTUs using the average neighbor algorithm with the 97% sequence similarity criterion. For each OTU, taxonomy was assigned using the naïve Bayesian classifier algorithm [25]. Low abundance and erroneous OTUs (abundance ≤ 0.005% of total abundance) were filtered out as described previously [32]. Furthermore, to lower the bias due to variation in sequence numbers across the samples, the OTU table was normalized by subsampling to equal sequence numbers (15,613) per sample. Rarefaction curves show that full richness of a community has been reached showing a good sequencing depth (Additional file 1: Fig. S1). OTUs with the same taxonomic identification were grouped into same genera for further analysis at the genus level, and taxa with relative abundance < 0.005% were grouped under the “others” category.

### Statistical analysis

The species richness and species diversity index (Shannon diversity index) were calculated using the vegan package in R statistical platform (version 3.5.3). Abundance and diversity figures representing the genus and phylum level were generated in GraphPad Prism version 8.4.1 for Windows (GraphPad Software, San Diego, CA, USA). *In silico* removal of likely endosymbionts was conducted, and OTU abundance was normalized accordingly. Initially, Pearson *r* correlation was used to calculate the correlation between CFU abundance and bacterial abundance by the culture-independent method and to statistically compare agonistic patterns among specific OTUs. Then, we conducted Spearman *r* correlation testing (non-parametric) for accurate representation of non-normally distributed data. Statistical analyses and plots were generated using GraphPad Prims version 8.4.1.

### OTU downstream analysis

Phylogenetic analysis using the sequences obtained by the 97% sequence identity criterion and an additional analysis and at 99% sequence identity were conducted to construct phylogenetic trees to search for endosymbiont genotypes and unclassified taxa. Reference sequences were obtained from the Genbank database at NCBI, and phylogenetic trees were generated in MEGA-X: Molecular Evolutionary Genetics Analysis version 10.1.6 [33]. Alignments were generated using the multiple sequence comparison by log-expectation (MUSCLE) and the UPGMA clustering methods. Trees were generated by the maximum likelihood (ML) method with 500 bootstrapping (Additional file 2: Fig. S2), which was also supported by neighbor joining (NJ), and unweighted pair group methods with the arithmetic mean (UPGMA).

## Results

### Culture-dependent method

**Fig. 2:**
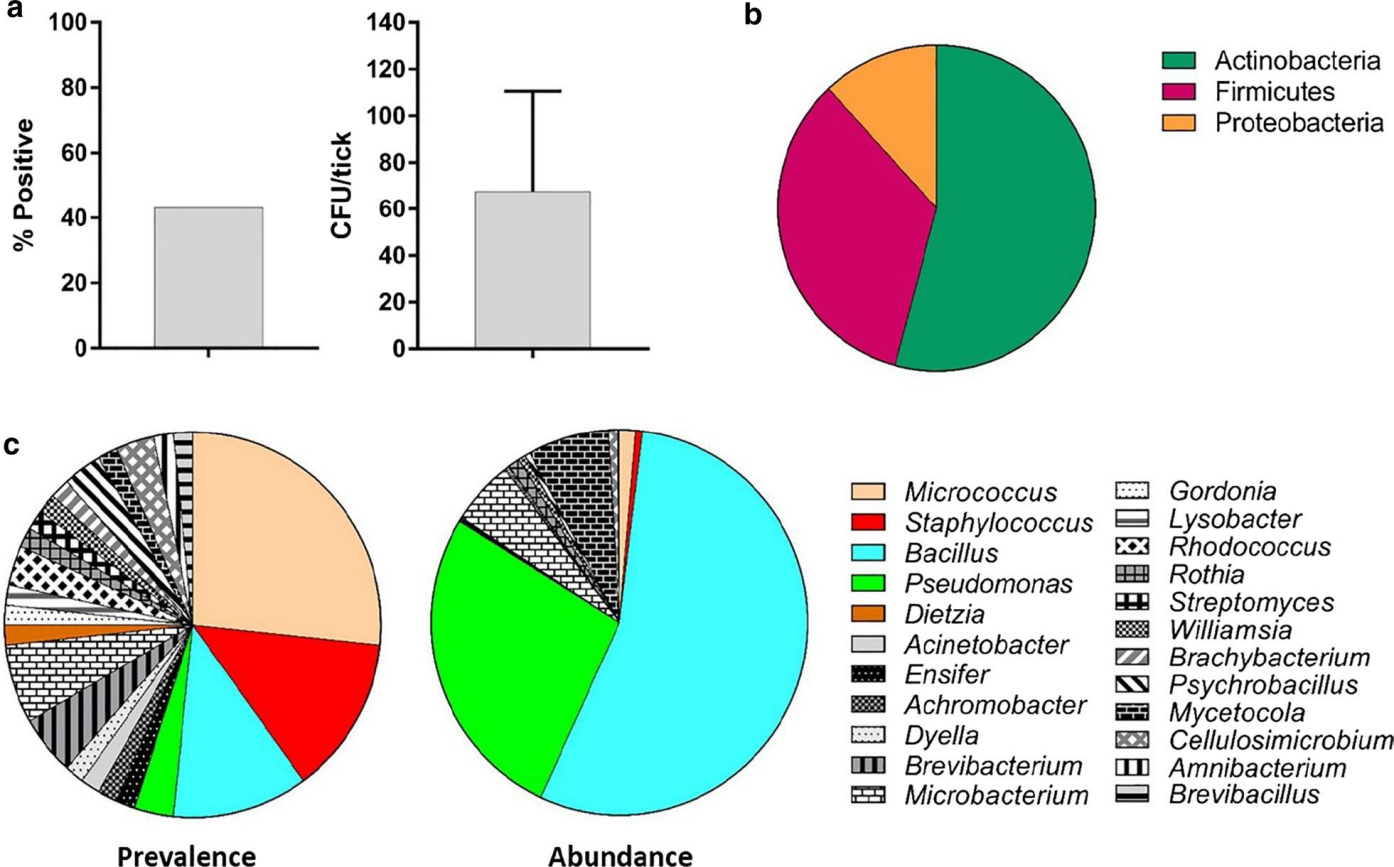
Bacteria cultured from *Ambylomma americanum*. **a** Prevalence (left) and abundance (right) in colony-forming units (CFU)/tick; **b** phylum abundance: Actinobacteria (54.2 %), Firmicutes (33.9 %), and Proteobacteria (11.9 %); **c** prevalence (in %) and abundance (in %) of bacterial genera (colored genera represent taxa also detected by culture-independent approach; black and white genera represent taxa identified by culture-dependent approach only)

We detected culturable bacteria from only 39.2% of ticks with abundance of 67.7 ± 42.8 CFU/tick (Fig. 2a). Sequencing of the 16S rRNA gene (~ 800 bp) revealed three bacterial phyla: Actinobacteria (54.2%), Firmicutes (33.9%), and Proteobacteria (11.9%) (Fig. 2b and Additional file 1: Fig. S1) with a heavy bias toward Gram-positive (88.1%) and catalase-positive (92.1%) taxa. A total of 45 species from 23 genera (Fig. 2c and Additional file 2: Fig. S2) were identified. The most prevalent genera were *Micrococcus* (26.6%), *Staphyloccocus* (13.3%), and *Bacillus* (11.6%) with the highest abundance of *Bacillus* (54.9%) and *Pseudomonas* (26.9%).

### Culture-independent method

**Fig. 3:**
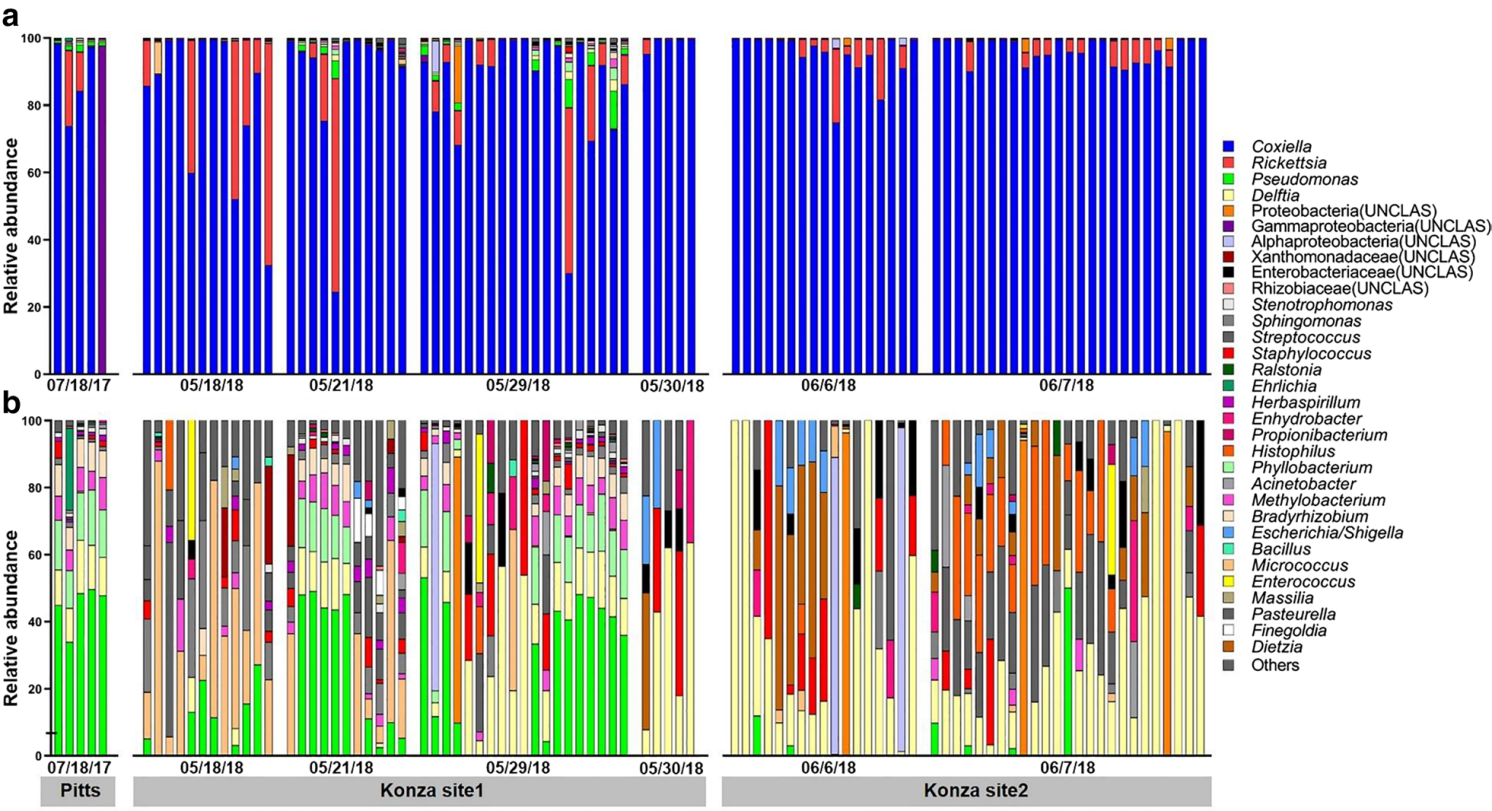
Relative abundance of bacterial genera obtained by the culture-independent method. **a** Relative abundance by genera of individual ticks; **b** relative abundance by genera after removal of endosymbionts (*Coxiella*, *Rickettsia* and Gammaproteobacteria). UNCLAS represent unclassified bacterial families or phyla

**Fig. 4:**
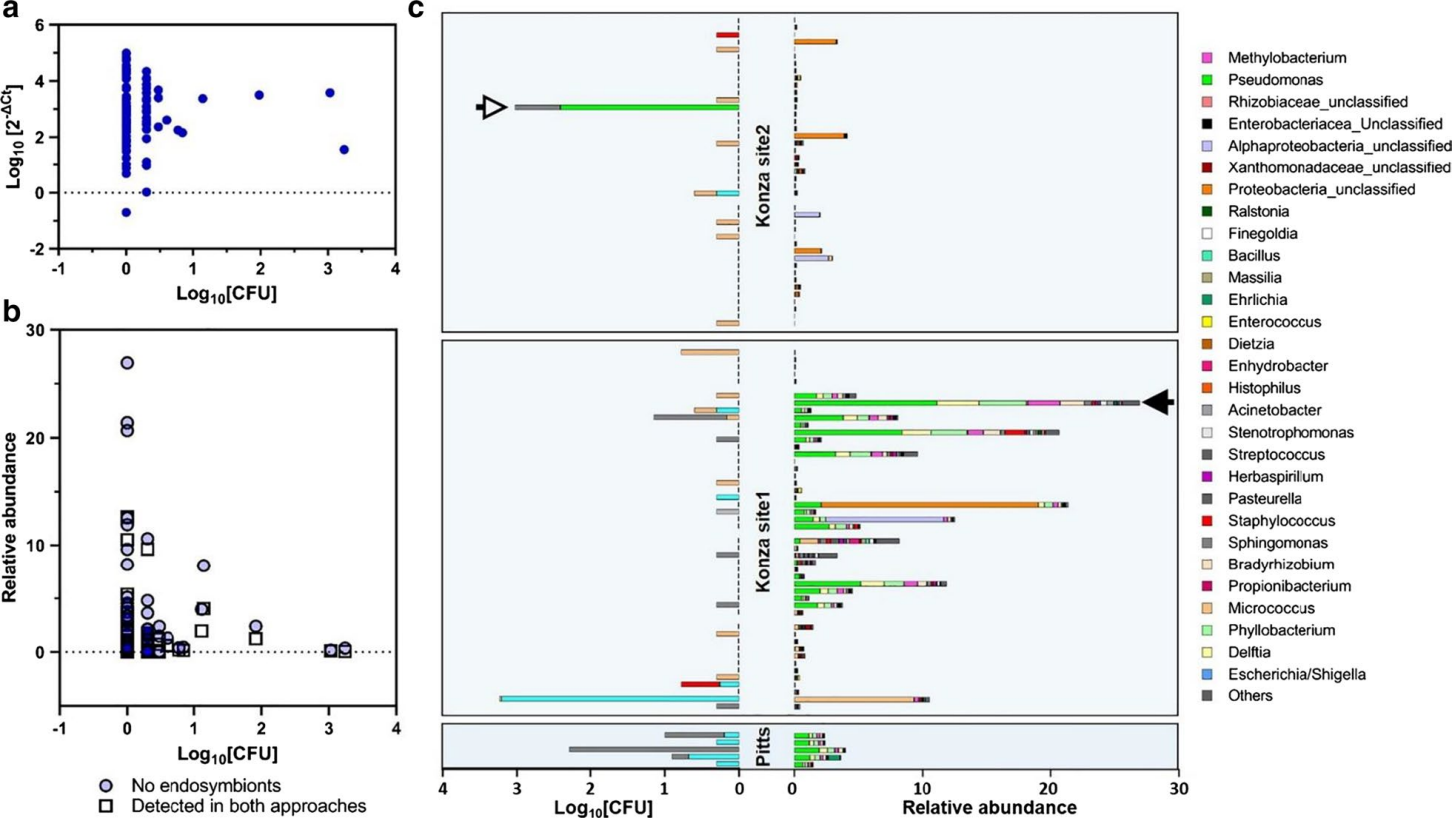
The 16S rRNA gene abundance by qPCR and its correlation to CFU abundance. **a** Scatterplot showing no correlation (*p* = 0.66) (between 16S rDNA abundance from qPCR and CFU abundance). Mean 16S copies per individual tick was 2.82 (log10[2^−ΔCT^]). **b** Scatterplot for correlation; circles represent total abundance of taxa excluding [*Coxiella*, *Rickettsia*, and Gammaproteobacteria (UNCLAS)] (*p* = 0.48); squares represent added abundance of taxa that were detected by culturing and identified in a culture-independent approach (*p* = 0.38). **c** CFU abundance diversity in individual ticks contrasted with the relative abundance of bacterial taxa from sequencing after exclusion of *Coxiella, Rickettsia*, and Gammaproteobacteria. Empty and black arrows point to tick samples 82 and 46 where no correlation between CFU abundance and HTS is observed

DNA extracts from 94 individual ticks resulted in a total of 236 OTUs using the average neighbor algorithm with the 97% sequence similarity criterion. After removal of taxa with very low relative abundance (< 0.005%) and grouping OTUs by the genus level, we obtained a total of 32 OTUs. Dominant genera were endosymbionts (*Coxiella* sp. and *Rickettsia* sp., 97.8% ± 0.4 of the reads per tick) (Fig. 3a). We performed *in silico* removal of the endosymbionts to better visualize the abundance of the extracellular bacteria. Our analysis revealed that the most abundant taxa (excluding endosymbionts) were typical soil- and plant-associated bacteria including *Pseudomonas*, *Bradyrhizobium*, *Micrococcus*, *Methylobacterium*, *Herbaspirillum*, *Acinetobacter*, and others (Fig. 3b). In an attempt to determine whether the high 16S rDNA abundance correlates with high CFU counts, we first measured the 16S rDNA abundance (by quantitative PCR) of each individual tick. This analysis included all 16S rDNA present within each individual. The variation of 16S rDNA was very high (six orders of magnitude for the largest difference). The average of 16S rDNA copy number in each tick was 2.82 (log_10_[2^−ΔCT^]), presenting ~ 630× more 16S copy number compared to the tick single-copy gene *V-ATPase subunit C*. The major bacterial species, *Coxiella*, is known to have one copy of 16S, allowing direct conversion of the 16S copy number to the bacterial number, although the bacterial 16S copy number varies depending on the species. Therefore, we conclude that there are approximately ~ 630 times more bacteria than the tick cell numbers with large variations among individual ticks. No correlation was found between the CFU abundance in the culture and the 16S rRNA qPCR abundance (*r* = 0.045; *p* = 0.66) (Fig. 4a).

In addition, no correlation was found between CFU abundance and relative abundance of non-endosymbiotic taxa and taxa identified by both approaches (culture-dependent and independent methods); (*r* = 0.07; *p* = 0.48 and *r* = 0.09; *p* = 0.38, respectively) (Fig. 4b). This suggests that large amounts of 16S rDNA from endosymbionts masked other OTUs in most cases. Several ticks with high CFU counts had low relative abundance of culturable taxa from sequencing (Fig. 4c). For example, tick 82 (white arrow in Fig. 4c) with the high CFU abundance of *Pseudomonas* sp. did not have any detectable *Pseudomonas* sp. reads. Likewise, high abundance of *Pseudomonas*, *Bradyrhizobium*, *Methylobacterium*, *Streptococcus*, and *Phyllobacterium* in tick 46 (black arrow in Fig. 4c) was not matched in CFUs from the culturing approach.

**Fig. 5:**
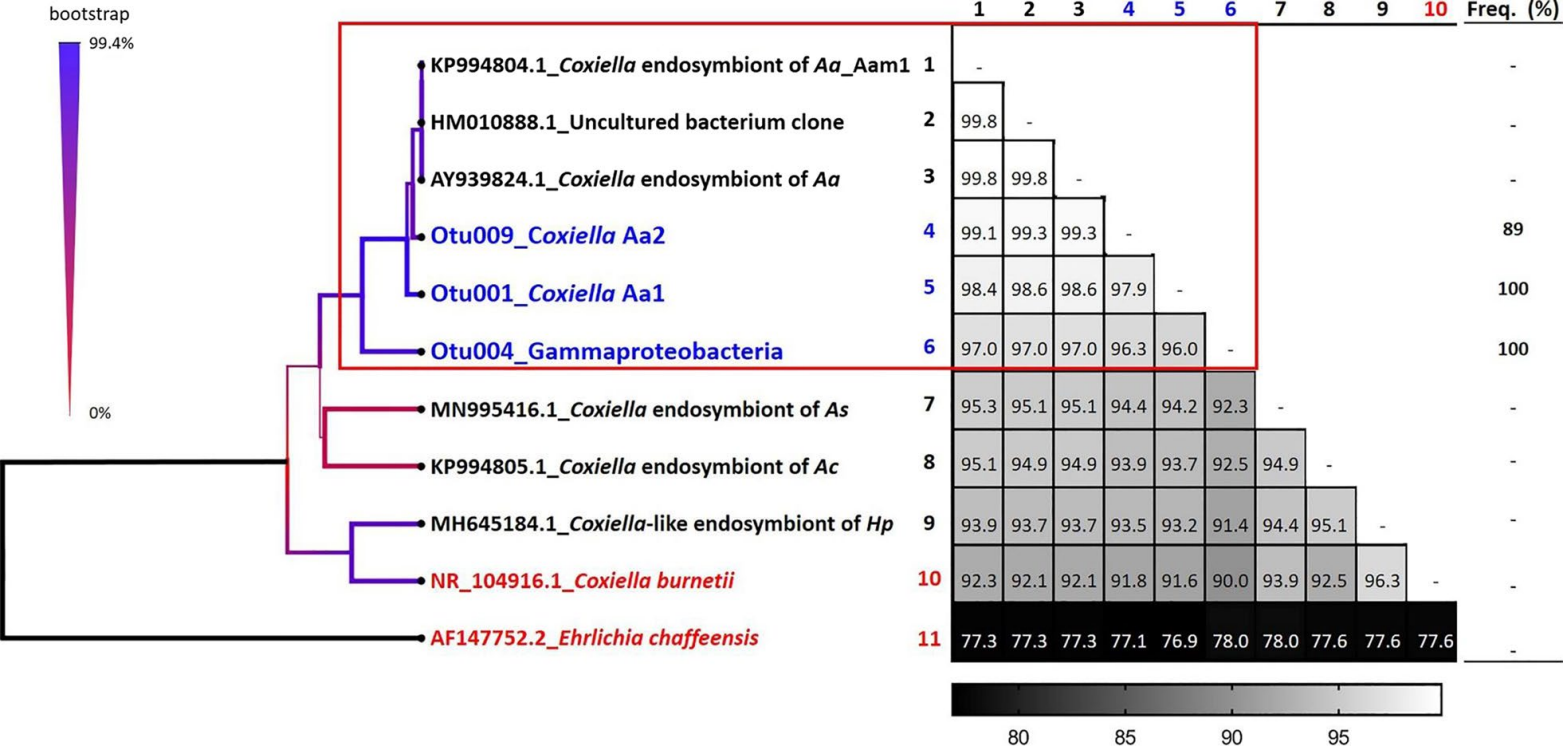
Phylogenetic tree showing the relationship among *Coxiella* endosymbionts, Gammaproteobacteria UNCLAS, and *Coxiella burnetii* of *Amblyomma* sp. OTUs obtained in this study (blue), references from data sets of known endosymbionts (black), and pathogens (red) of the *A. americanum.* UPGMA phylogram with bootstrap test (500 replicates) depict bootstrap values as a weighted line and in blue and red color scale (values > 70 are shown in blue and purple). Matrix table shows % identity of each taxon against the other. Frequency (%) shows the frequency at which the taxa were found in the tick samples

Phylogenetic trees showed that unclassified (UNCLAS) Gammaproteobacteria clustered with endosymbionts of *Amblyomma* sp. We also detected two *Coxiella* genotypes (genotype Aa1 and Aa2, with 98.6 and 99.3% identity, respectively to *Coxiella* endosymbiont (AY939824.1) (Fig. 5). *Coxiella* Aa1 was dominant (100% frequency) and abundant (88.3%), while the *Coxiella* Aa2 was less frequent (89%) and with very low abundance (0.1%) (Fig. 5).

**Fig. 6:**
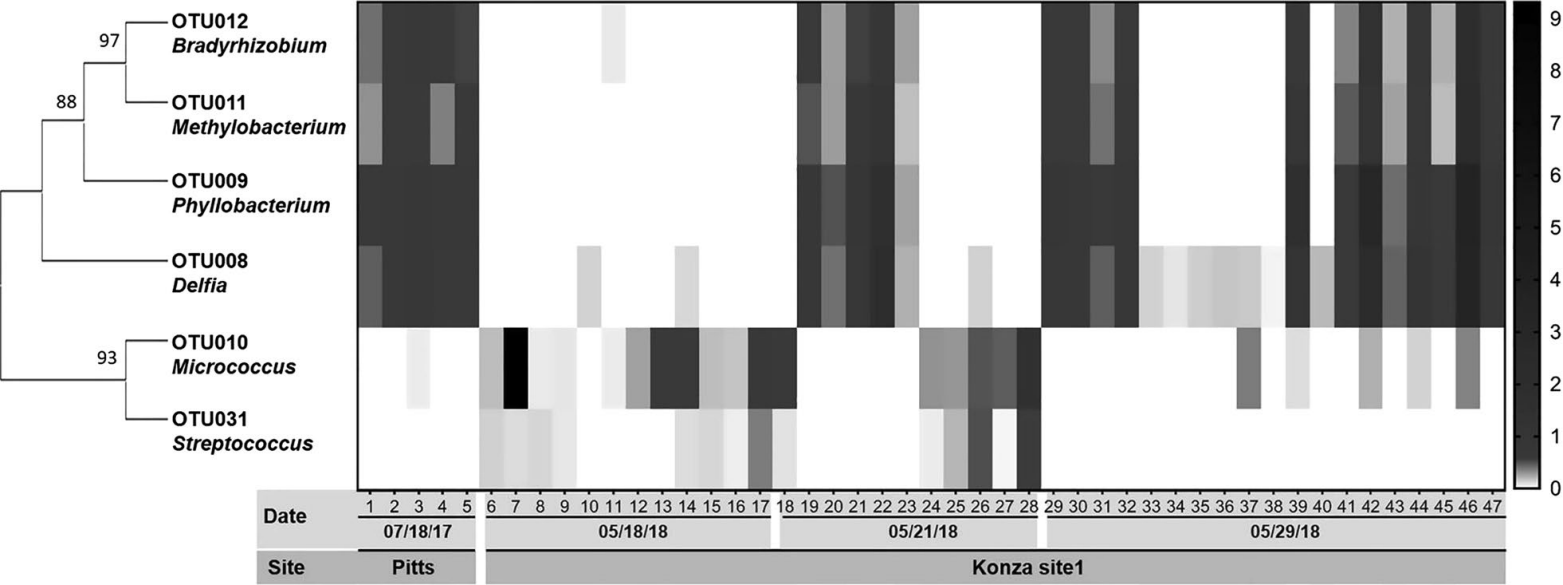
Heat map and phylogeny of taxa with potential agonist and antagonistic relationships in *Amblyomma americanum* (*n* = 47). Absence in white and presence in gray to black. Phylogeny is based on the maximum likelihood clustering with 500 bootstraps

We also found distribution patterns of bacterial taxa implying antagonistic and agonistic relationships among certain bacteria. A closely related taxonomic group, *Delfia*, *Phyllobacterium*, *Methylobacterium*, and *Bradyrhizobium* (OTUs 8, 9, 11 and 12) were found in the same individual ticks, while *Micrococcus* sp. (OTU10) and *Streptococcus* (OTU31) were also found together, but in different individual ticks (Fig. 6). This potential antagonistic distribution pattern was found in 47 ticks (53% of total). These two groups appeared to be in a mutually exclusive manner in each individual tick through our manual search. However, only the agonistic taxa (OTUs 8, 9, 11, and 12) were found to be statistically correlated to each other (Pearson’s correlation coefficients *r* = 0.95–0.98), and no significant correlation was observed between OTU10 and OTU31 (*r* = 0.34, *p* = 0.052).

## Discussion

Data from our culture-dependent approach clearly show that the midgut microbiome of *A. americanum* is very poor with low abundance and no core bacterial community compared to those of other blood-feeding arthropods [34]. This is in agreement with recent studies on the gut microbiome of other tick species including *Ixodes scapularis* [35] and *Ixodes ricinus* [36]. The majority of the bacterial taxa were representative of soil- and plant-associated bacteria. This is not surprising since *A. americanum* has been shown to actively ingest liquid water from the environment to recuperate the imminent water losses occurring through excretion mechanisms [37–39], and it this therefore likely that these bacteria were ingested along with water [10]. The soil and plant origin of the midgut bacteria was also suggested for *I. ricinus* [10, 40]. It is however intriguing that the majority of isolates were gram positive and catalase positive, indicating that these attributes likely play a role in bacteria resisting tick epithelial immunity responses including the action of dual oxidases maintaining tick bacterial homeostasis [41]. Nevertheless, even for these taxa, the abundance was very low, and in more than half of the tick samples no culturable bacteria were found.

The culture-independent approach revealed that most of the tick microbiome is composed of known endosymbiotic bacteria with *Coxiella* sp. as the dominant taxon followed by *Rickettsia* sp. Other bacterial community members were low in abundance and also dominated by bacteria typical for the soil and plant environment, as previously suggested [42], and this corroborates the results from the culturing approach. Overall, our findings contrast previous reports of an abundant bacterial community in ticks reporting several hundreds of OTUs [13, 19, 20] using similar culture-independent methods. It is possible that the rich and abundant bacterial community detected in other culture-independent studies in ticks was a result of contamination by bacteria from the tick surface as recently suggested by Binetruy et al. [36]. That study showed that surface sterilization methods significantly impact the internal bacterial community composition. We have used a thorough surface sterilization protocol with sodium hypochlorite and ethanol as the most effective method of sterilization [36], and this very likely avoided any major contamination from the tick surface.

Standardizing optimal conditions for detection and isolation of all culturable bacteria is challenging [43], and although our culture-dependent approach is limited to culturable aerobic and microaerophilic bacteria on a broad-spectrum nutrient agar, we believe it provides the sufficient evidence to show limited abundance of extracellular bacteria in the tick midgut. Many culturable taxa that were also commonly identified in the culture-independent approach were Gram negative such as *Pseudomonas*, *Delftia*, and unidentified *Proteobacteria*. The culturable taxa captured in only 16S rDNA sequencing, but not in the culture of the same homogenates, are likely a result of amplification of DNA from lysed cells or DNA of cells in viable but not culturable state [44]. Clearly, the lack of correlation between results of the culture and culture-independent (Miseq and qPCR) approaches indicates the limits of both approaches. Our culture-independent approach results revealed that > 95% of bacteria represented non-culturable endosymbionts, which is in accordance with other tick microbiome studies [13–16, 43]. In our study, we used a 0.005% cutoff for the OTU downstream analysis, which is very low for microbial community studies and allowed us to show bacterial taxa with very low abundance, some of which were also detected by culturing. Overall, it is very likely that many bacteria, especially those with low abundance, were masked by the dominant endosymbionts *Coxiella* and *Rickettsia*, which could explain why some of the isolates identified using the culture-dependent approach were not detected in the culture-independent approach.

Phylogenetic analysis revealed two different *Coxiella* genotypes and an unidentified Gammaproteobacterium (OTU 004), all of which were closely related to known the *Coxiella* endosymbiont of *A. americanum.* Since all three were detected in high frequency, this greatly reduces the likelihood of artifacts including sequencing errors. Nonetheless, more in-depth analysis including sequencing of the entire 16S rRNA gene is needed [45] to uncover the phylogeny of these symbionts.

We also detected potential agonist and antagonist relationships among specific bacterial genera by a manual search. Specifically, *Micrococcus* sp. and *Streptococcus* sp. were absent when taxa *Delfia*, *Phyllobacterium*, *Methylobacterium*, and *Bradyrhizobium* were present, and *vice versa*. The statistical analysis revealed strong correlation among the agonist taxa; however, no significant antagonistic effect was shown with our statistical analysis. The antagonist phenomenon is common among bacteria in other animals [46], and it could also have biological significance for the vector competence of *A. americanum* for pathogens such *Francisella tularensis* and *Anaplasma* sp. as proposed for the midgut bacteria of *I. scapularis* and *Borrelia burgdorferi* [47].

Shannon’s diversity index and species richness varied across samples (0.269 ± 0.03 and 9.04 ± 0.44; respectively); however, the overall diversity and species richness were significantly lower compared to those in other studies on *I. scapularis* [48] where the overall Shannon index was between 1.0 and 2.5 and the overall species richness between 10.0 and 20.0. This variation can be attributed to the difference in tick species, tick distribution, and potentially the microhabitat, which could influence tick microbial community [40, 49, 50].

In conclusion, the microbiome of *A. americanum* is dominated by endosymbionts, and these are likely more diverse than believed previously. The midgut bacterial community of this tick species is poor without a core microbiome. Nevertheless, there are several culturable bacterial taxa that could be used for further experimental studies addressing: (1) whether these are transient or capable of midgut colonization, (2) how midgut epithelial immunity maintains such a low bacterial abundance, and (3) the role these bacteria play in the vector competence of *A. americanum* for pathogens.

## Supplementary Information

**Additional file 1: Figure S1.** Rarefaction curves of individual tick samples. The OTU table was rarefied to equal sequence numbers (15,613) per sample. Curves are color coded by location: red: Konza-1; cyan: Konza-2; blue: Pittsburg.

**Additional file 2: Figure S2.** Maximum likelihood tree of the 16S rDNA of bacterial isolates from *Amblyomma americanum*. The tree with the highest log likelihood is shown. The percentage of trees in which the associated taxa clustered together is shown next to the branches (500 replicates). Initial trees for the heuristic search were obtained automatically by applying neighbor joining and BioNJ algorithms to a matrix of pairwise distances estimated using the maximum composite likelihood (MCL) approach and then selecting the topology with a superior log likelihood value. This analysis involved 45 nucleotide sequences. There were a total of 785 positions in the final dataset. Colors of the tree branches indicate the phyla: Actinobacteria (green); Firmicutes (magenta); Proteobacteria (red).

## Data Availability

Not applicable.

## References

[1] Brites-Neto J, Duarte KM, Martins TF (2015). Tick-borne infections in human and animal population worldwide. Vet World.

[2] Dantas-Torres F, Chomel BB, Otranto D (2012). Ticks and tick-borne diseases: a one health perspective. Trends Parasitol.

[3] de la Fuente J, Estrada-Pena A, Venzal JM, Kocan KM, Sonenshine DE (2008). Overview: ticks as vectors of pathogens that cause disease in humans and animals. Front Biosci.

[4] CDC. Map of established Amblyomma americanum tick populations in the United States 2019. 2019.

[5] Tokarz R, Sameroff S, Tagliafierro T, Jain K, Williams SH, Cucura DM (2018). Identification of novel viruses in *Amblyomma americanum*, *Dermacentor variabilis*, and *Ixodes scapularis* ticks. mSphere.

[6] Sayler KA, Loftis AD, Beatty SK, Boyce CL, Garrison E, Clemons B (2016). Prevalence of tick-borne pathogens in host-seeking *Amblyomma americanum* (Acari: Ixodidae) and *Odocoileus virginianus* (Artiodactyla: Cervidae) in Florida. J Med Entomol.

[7] Mixson TR, Campbell SR, Gill JS, Ginsberg HS, Reichard MV, Schulze TL (2006). Prevalence of *Ehrlichia*, *Borrelia*, and *Rickettsial* agents in *Amblyomma americanum* (Acari: Ixodidae) collected from nine states. J Med Entomol.

[8] Crispell G, Commins SP, Archer-Hartman SA, Choudhary S, Dharmarajan G, Azadi P, et al. Discovery of alpha-gal-containing antigens in North American tick species believed to induce red meat allergy. Front Immunol. 2019;10:1664–3224 (Electronic):1–16; 10.3389/fimmu.2019.01056. 10.3389/fimmu.2019.01056 PMC6533943 31156631

[9] Park YAO, Kim D, Boorgula GD, De Schutter KAO, Smagghe GAO, Šimo L (2019). Alpha-gal and cross-reactive carbohydrate determinants in the *N*-glycans of salivary glands in the lone star tick, *Amblyomma americanum*. Vaccines.

[10] Bonnet SI, Binetruy F, Hernandez-Jarguin AM, Duron O (2017). The tick microbiome: why non-pathogenic microorganisms matter in tick biology and pathogen transmission. Front Cell Infect Microbiol.

[11] de la Fuente J, Antunes S, Bonnet S, Cabezas-Cruz A, Domingos AG, Estrada-Pena A (2017). Tick-pathogen interactions and vector competence: identification of molecular drivers for tick-borne diseases. Front Cell Infect Microbiol.

[12] Narasimhan S, Fikrig E (2015). Tick microbiome: the force within. Trends Parasitol.

[13] Clay K, Klyachko O, Grindle N, Civitello D, Oleske D, Fuqua C (2008). Microbial communities and interactions in the lone star tick, *Amblyomma americanum*. Mol Ecol.

[14] Heise SR, Elshahed MS, Little SE (2010). Bacterial diversity in *Amblyomma americanum* (Acari: Ixodidae) with a focus on members of the genus *Rickettsia*. J Med Entomol.

[15] Jasinskas A, Zhong J, Barbour AG (2007). Highly prevalent *Coxiella* sp. bacterium in the tick vector *Amblyomma americanum*. Appl Environ Microbiol.

[16] Klyachko O, Stein BD, Grindle N, Clay K, Fuqua C (2007). Localization and visualization of a *Coxiella*-type symbiont within the lone star tick, *Amblyomma americanum*. Appl Environ Microbiol.

[17] Narasimhan S, Rajeevan N, Liu L, Zhao YO, Heisig J, Pan J (2014). Gut microbiota of the tick vector *Ixodes scapularis* modulate colonization of the Lyme disease spirochete. Cell Host Microbe.

[18] Trout Fryxell RT, DeBruyn JM (2016). Correction: The microbiome of Ehrlichia-infected and uninfected lone star ticks (*Amblyomma americanum*). PLoS ONE.

[19] Williams-Newkirk AJ, Rowe LA, Mixson-Hayden TR, Dasch GA (2014). Characterization of the bacterial communities of life stages of free living lone star ticks (*Amblyomma americanum*). PLoS ONE.

[20] Menchaca AC, Visi DK, Strey OF, Teel PD, Kalinowski K, Allen MS (2013). Preliminary assessment of microbiome changes following blood-feeding and survivorship in the *Amblyomma americanum* nymph-to-adult transition using semiconductor sequencing. PLoS ONE.

[21] Greay TL, Gofton AW, Paparini A, Ryan UM, Oskam CL, Irwin PJ (2018). Recent insights into the tick microbiome gained through next-generation sequencing. Parasites Vectors.

[22] Hernandez-Jarguin A, Diaz-Sanchez S, Villar M, de la Fuente J (2018). Integrated metatranscriptomics and metaproteomics for the characterization of bacterial microbiota in unfed *Ixodes ricinus*. Ticks Tick Borne Dis.

[23] Turner S, Pryer KM, Miao VP, Palmer JD (1999). Investigating deep phylogenetic relationships among cyanobacteria and plastids by small subunit rRNA sequence analysis. J Eukaryot Microbiol.

[24] Altschul SF, Gish W, Miller W, Myers EW, Lipman DJ (1990). Basic local alignment search tool. J Mol Biol.

[25] Wang Q, Garrity GM, Tiedje JM, Cole JR (2007). Naive Bayesian classifier for rapid assignment of rRNA sequences into the new bacterial taxonomy. Appl Environ Microbiol.

[26] Cole JR, Chai B, Farris RJ, Wang Q, Kulam SA, McGarrell DM, Garrity GM (2005). The Ribosomal Database Project (RDP-II): sequences and tools for high-throughput rRNA analysis. Nucleic Acids Res.

[27] Yarza P, Caporaso CL, Walters WA, Berg-Lyons D, Lozupone CA, Turnbaugh PJ (2011). Global patterns of 16S rRNA diversity at a depth of millions of sequences per sample. PNAS.

[28] Schloss PD, Westcott SL, Ryabin T (2009). Introducing mothur: Open-source, platform-independent, community-supported software for sescribing and comparing microbial communities. Appl Environ Microbiol.

[29] Yilmaz P, Parfrey LW, Yarza P (2014). The SILVA and “All-species living tree project (LTP)” taxonomic frameworks. Nucleic Acids Res.

[30] Needleman SB, Wunsch CD (1970). A general method applicable to the search for similarities in the amino acid sequence of two proteins. J Mol Biol..

[31] Edgar RC, Haas BJ, Clemente JC, Quince C, Knight R (2011). UCHIME improves sensitivity and speed of chimera detection. Bioinformatics..

[32] Bokulich NA, Subramanian S, Faith JJ, Gevers D, Gordon JI, Knight R (2013). Quality-iltering vastly improves diversity estimates from Illumina amplicon sequencing. Nat Methods..

[33] Kumar S, Stecher G, Li M, Knyaz C, Tamura K (2018). MEGA X: molecular evolutionary genetics analysis across computing platforms. Mol Biol Evol..

[34] Strand MR. Chapeter 11 The gut microbiota of mosquitoes diversity and function. In: Arthropod vector: controller of disease transmission, vol. 1. London: Academic Press; 2017. p. 185–99.

[35] Ross BD, Hayes B, Radey MC, Lee X, Josek T, Bjork J (2018). *Ixodes scapularis* does not harbor a stable midgut microbiome. ISME J..

[36] Binetruy F, Dupraz M, Buysse M, Duron O (2019). Surface sterilization methods impact measures of internal microbial diversity in ticks. Parasites Vectors..

[37] Maldonado-Ruiz LP, Park Y, Zurek L. Liquid water intake of the lone star tick, *Amblyomma americanum*: implications for tick survival and management. 2020. https://europepmc.org/articles/PMC7138852. 10.1038/s41598-020-63004-9 PMC7138852 32265527

[38] Kim D, Maldonado-Ruiz P, Zurek L, Park Y (2017). Water absorption through salivary gland type I acini in the blacklegged tick Ixodes scapularis. PeerJ..

[39] Kim D, Simo L, Vancova M, Urban J, Park Y (2019). Neural and endocrine regulation of osmoregulatory organs in tick: recent discoveries and implications. Gen Comp Endocrinol..

[40] Aivelo T, Norberg A, Tschirren B (2019). Bacterial microbiota composition of *Ixodes ricinus* ticks: the role of environmental variation, tick characteristics and microbial interactions. PeerJ.

[41] Yang X, Smith AA, Williams MS, Pal U (2014). A dityrosine network mediated by dual oxidase and peroxidase inluences the persistence of Lyme disease pathogens within the vector. J Biol Chem..

[42] Stewart PE, Bloom ME (2020). Sharing the ride: Ixodes scapularis symbionts and their interactions. Front Cell Infect Microbiol..

[43] Lagier J-C, Dubourg G, Million M, Cadoret F, Bilen M, Fenollar F (2018). Culturing the human microbiota and culturomics. Nat Rev Microbiol..

[44] Li L, Mendis N, Trigui H, Oliver JD, Faucher SP (2014). The importance of the viable but non-culturable state in human bacterial pathogens. Front Microbiol.

[45] Johnson JS, Spakowicz DJ, Hong BY, Petersen LM, Demkowicz P, Chen L (2019). Evaluation of 16S rRNA gene sequencing for species and strain-level microbiome analysis. Nat Commun..

[46] García-Bayona L, García-Bayona LE (2018). Bacterial antagonism in host-associated microbial communities. Science.

[47] Chou S, Daugherty MD, Peterson SB, Biboy J, Yang Y, Jutras BL (2014). Transferred interbacterial antagonism genes augment eukaryotic innate immune function. Nature.

[48] Hernandez-Jarguin A, Diaz-Sanchez S, Villar M, de la Fuente J (2018). Integrated metatranscriptomics and metaproteomics for the characterization of bacterial microbiota in unfed *Ixodes ricinus*. Ticks Tick Borne Dis..

[49] Thapa S, Zhang Y, Allen MS (2019). Effects of temperature on bacterial microbiome composition in ticks. MicrobiologyOpen..

[50] Estrada-Peña A, Cabezas-Cruz A, Pollet T, Vayssier-Taussat M, Cosson J-F (2018). High throughput sequencing and network analysis disentangle the microbial communities of ticks and hosts within and between ecosystems. Fron Cell Infect Microbiol..

