# Supplementary figures and images for: The bacterial community of the lone star tick (Amblyomma americanum)
Source: Parasit Vectors. 2021 Jan 14;14:49. doi: 10.1186/s13071-020-04550-z (PMC7807426; doi:10.1186/s13071-020-04550-z)

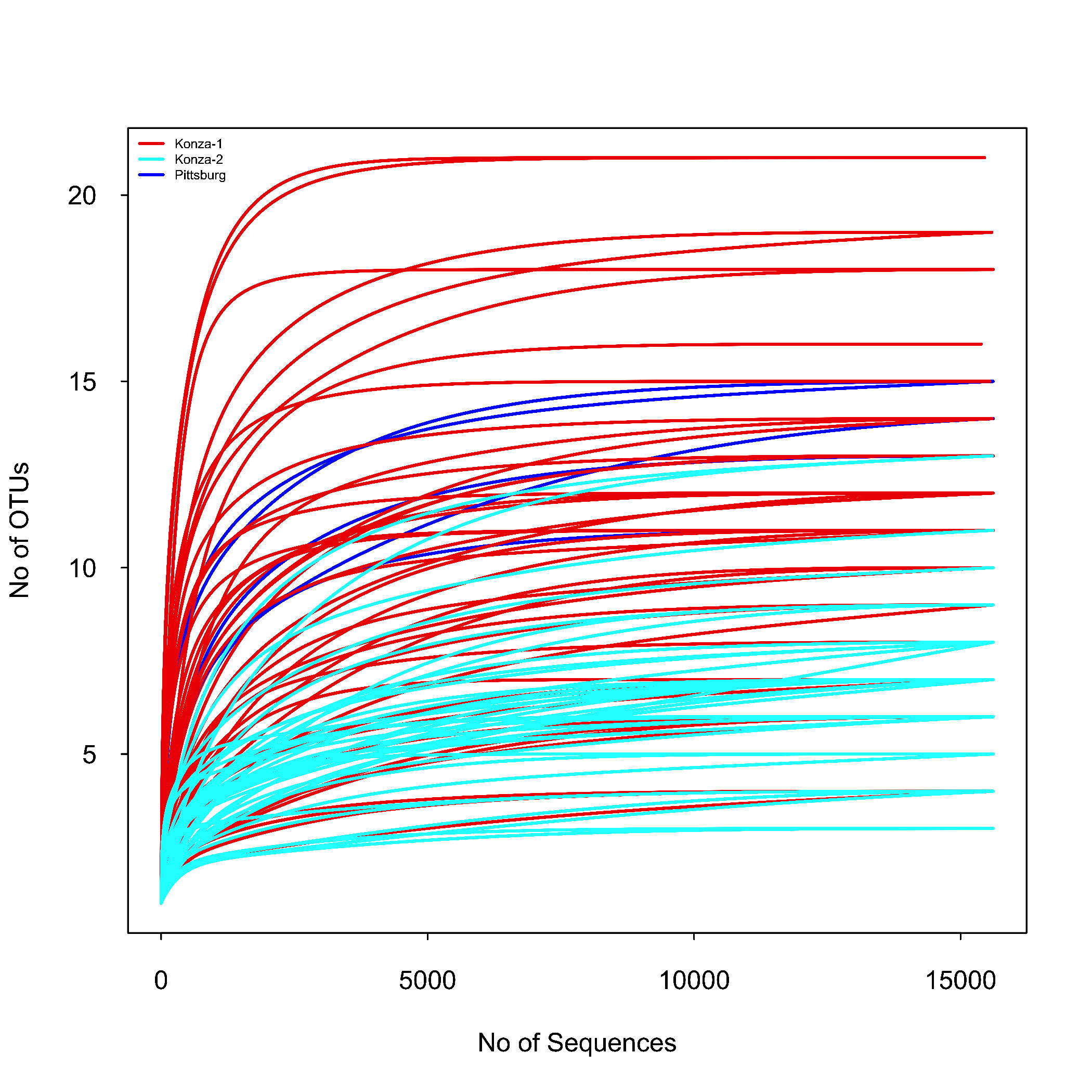

Supplement: Supplementary file 1 — Additional file 1: Figure S1. Rarefaction curves of individual tick samples. The OTU table was rarefied to equal sequence numbers (15,613) per sample. Curves are color coded by location: red: Konza-1; cyan: Konza-2; blue: Pittsburg. [file 13071_2020_4550_MOESM1_ESM.jpg]

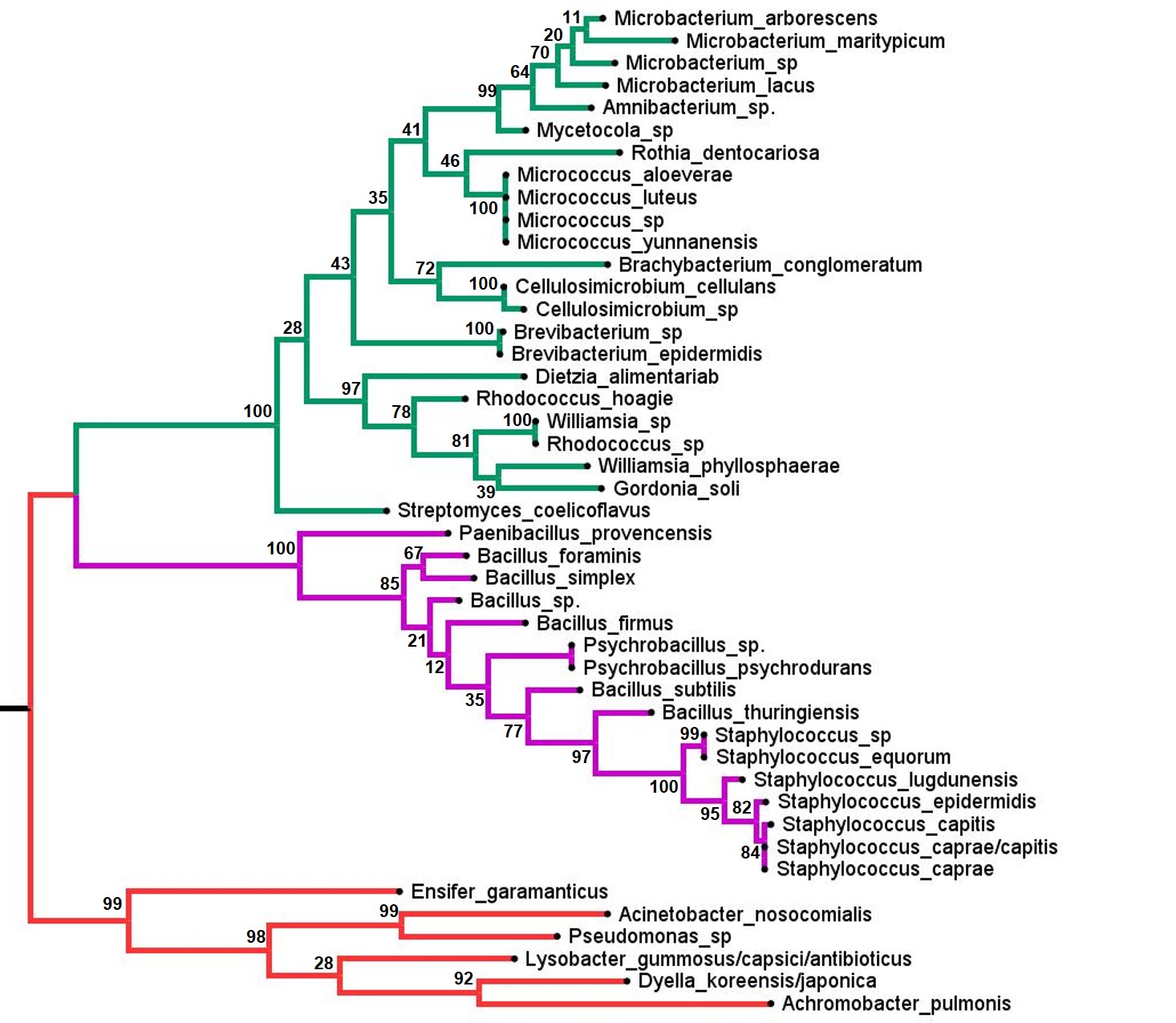

Supplement: Supplementary file 2 — Additional file 2: Figure S2. Maximum likelihood tree of the 16S rDNA of bacterial isolates from Amblyomma americanum. The tree with the highest log likelihood is shown. The percentage of trees in which the associated taxa clustered together is shown next to the branches (500 replicates). Initial trees for the heuristic search were obtained automatically by applying neighbor joining and BioNJ algorithms to a matrix of pairwise distances estimated using the maximum composite likelihood (MCL) approach and then selecting the topology with a superior log likelihood value. This analysis involved 45 nucleotide sequences. There were a total of 785 positions in the final dataset. Colors of the tree branches indicate the phyla: Actinobacteria (green); Firmicutes (magenta); Proteobacteria (red). [file 13071_2020_4550_MOESM2_ESM.jpg]
